# Different fungal and bacterial pathogen infections alter the grapevine microbiome and phenolic profiles in a localized and specific manner

**DOI:** 10.3389/fpls.2026.1838241

**Published:** 2026-06-16

**Authors:** Claudia Castro, Nalong Mekdara, Frank Harmon, Devin Coleman-Derr, Christopher M. Wallis

**Affiliations:** 1 Plant Gene Expression Center, United States Department of Agriculture- Agricultural Research Service, Albany, CA, United States; 2 Crop Diseases, Pests and Genetics Research Unit, United States Department of Agriculture- Agricultural Research Service, San Joaquin Valley Agricultural Sciences Center, Parlier, CA, United States

**Keywords:** 16S rRNA, fungal trunk disease, metabolomics, Pierce’s disease, *Vitis vinifera*

## Abstract

**Introduction:**

Plant pathogens pose a critical threat to global agriculture by significantly reducing crop productivity through the negative impact on plant physiology and associated microbial communities. Grapevines (*Vitis vinifera*), a high-value crop worldwide, are highly susceptible to a range of bacterial and fungal vascular pathogens. In this study, we investigated the effects of five major grapevine pathogens—*Diplodia seriata*, *Eutypa lata*, *Neofusicoccum parvum*, *Phaeoacremonium minimum*, and *Xylella fastidiosa*—on host microbiome composition and phenolic secondary metabolite profiles across multiple plant tissues.

**Materials and methods:**

For grapevines infected with each of the five pathogens, a combination of 16S rRNA and Internal Transcribed Spacer (ITS) sequencing was performed alongside high-performance liquid chromatography analysis. Generated data were compared to observe if changes in host chemistry caused by infection from one of the pathogens could be associated with shifts in microbial communities both around and away from the initial infection sites.

**Results:**

We found that pathogen infection induced significant pathogen-specific alterations in both bacterial and fungal communities, predominantly at the inoculation site. Additionally, infections triggered localized changes in phenolic compounds, especially stilbenoids, consistent with host defense responses. Notably, fungal pathogens broadly disrupted bacterial communities, while *X. fastidiosa* had a more limited and distal effect.

**Discussion:**

Our findings highlight distinct microbiome and metabolic signatures associated with each pathogen and underscore the importance of examining different host tissues in studying plant–microbiome–pathogen interactions. These insights contribute to a systems-level understanding of grapevine disease ecology and may inform future strategies for monitoring and controlling pathogen spread in perennial crops such as grapevines.

## 1 Introduction

Plant pathogens significantly decrease crop production by ~20% to 30% each year, resulting in economic losses estimated to be in the billions of dollars worldwide (Savary et al., 2019; Chaloner et al., 2021; Singh et al., 2023). Under favorable environmental conditions, plant pathogens may spread rapidly to cause a disease outbreak. Likewise, when environmental conditions are favorable and a pathogen is present, a large population of pathogen-vectoring insects would likely result in a disease outbreak. Even when not physically present throughout the entire plant, pathogens can have a strong and swift influence over the physiology of non-colonized distal parts of their host. This is through both indirect effects, such as plant immune responses, tissue repair mechanisms, or changes in host physiological capacity due to tissue losses, and direct effects, such as the dissemination of virulence factors (e.g., cell wall-degrading enzymes, protein effectors, and phytotoxins) (Massonnet et al., 2017; Klessig et al., 2018; Wallis and Galarneau, 2020; Castro et al., 2023). Studying the impact of these pathogens across plant tissues (i.e., roots, leaves, and stems) is important for understanding the infection process from a holistic view, which will in turn aid in developing solutions for monitoring and controlling disease (Wallis and Chen, 2012; Wallis et al., 2013, Wallis et al., 2022).

Disease-causing bacteria, oomycetes, and fungi are a part of the microbiome that is in and around plant tissues (Vandenkoornhuyse et al., 2015; Teixeira et al., 2019; Ginnan et al., 2020). As pathogens spread throughout the plant, interactions with native members of the microbiome can potentially support or thwart their infection of a variety of plant tissue types via positive (e.g., synergism) and negative interactions (direct and indirect competition) (Lamichhane and Venturi, 2015; Ritpitakphong et al., 2016; Berg and Koskella, 2018; Li et al., 2021; Vogel et al., 2021; Wallis et al., 2022). Changes in the plant microbiome by disease-causing agents have been documented in a wide variety of systems (Deyett et al., 2017; Purahong et al., 2018; Ginnan et al., 2020; Zhou et al., 2021). Improving our knowledge of how the microbiome responds to each specific pathogen is critical to developing effective and consistent solutions for disease mitigation (French et al., 2021; Ali et al., 2023; Xiong et al., 2023). Indeed, in order to move beyond the use of a single beneficial organism as a biological control to the use of an approach that involves an application of or methods to stimulate the natural production of an enhanced beneficial microbial community requires an understanding of how shifts in host physiology occur throughout the life of the plant (French et al., 2021). In woody perennial crops such as grapevines, this may mean persistent, long-term infections by stem-dwelling fungal or bacterial pathogens that would inevitably alter host physiology and, in turn, microbial communities, whether manipulated or not. Often, such multi-year infections have not been thoroughly considered in the use of microbial community manipulations to improve plant health, as the majority of existing studies have focused on annual plants (e.g., those studies highlighted by French et al., 2021).

Shifts in the plant microbiome that occur during pathogen infection are thought to be the result of both microbe–microbe interactions and triggered host defense responses that impact plant physiology (Kemen, 2014; Teixeira et al., 2019; Vogel et al., 2021). This includes a rewiring of gene expression that induces changes in plant hormone production, oxidative stress levels, pathogenesis-related proteins, and phenolic compounds (and other secondary metabolites) (Ngou et al., 2022; Yuan et al., 2021). The phenolic compounds may serve as antimicrobials and help strengthen and repair cell walls, among other roles (Wallis and Chen, 2012). Additionally, in woody plants, phenolic compounds play a significant role in the formation of lignin and suberin as a defense response to vascular pathogens and to repair damaged tissues (Rolshausen et al., 2008; Graça, 2010; Sun et al., 2013; Lewis, 2017; Rapicavoli et al., 2018; Serra and Geldner, 2022). Altered chemistry and the deployment of secondary metabolites within the cell, cell wall, and the apoplastic space in host tissues where endophytes reside can have a profound impact on microbiome composition and activity (Jones et al., 2019). Determining the extent to which pathogen infection leads to altered metabolism in plants, especially those that produce wood, will help us understand pathogenesis and plant–microbe interactions in greater depth.

Grapevines (*Vitis vinifera*) are a high-value specialty crop and are impacted by a wide variety of diseases caused by bacteria, fungi, viruses, and oomycetes, many of them being vascular pathogens. Fungal and bacterial vascular pathogens are among the most devastating microbes for vineyards across the world. These include fungal trunk pathogens *Diplodia seriata* (causal agent of *Botryosphaeria* dieback), *Eutypa lata* (causal agent of *Eutypa* dieback), *Neofusicoccum parvum* (causal agent of *Botryosphaeria* dieback), *Phaeoacremonium minimum* (causal agent of Esca or Petri disease), and the xylem-limited bacterium *Xylella fastidiosa* (causal agent of Pierce’s disease) (Gramaje et al., 2018; Kyrkou et al., 2018). Currently, there are no known *V. vinifera* cultivars that cannot become infected by *Xylella* (Gramaje et al., 2018; Kyrkou et al., 2018). However, different commercial grapevine cultivars and other *Vitis* species demonstrated improved tolerance or the ability to have reduced symptom progression to these pathogens when infected (Wallis et al., 2013; Wallis et al., 2020; Sinclair et al., 2025). While it is known that pathogens impact the microbiome and metabolism of the grape plants, most previous studies have focused on single or a few plant compartments (Wallis and Chen, 2012; Deyett et al., 2017; Massonnet et al., 2017; Deyett and Rolshausen, 2019; Galarneau et al., 2021). In this study, we hypothesized that *D. seriata*, *E. lata*, *N. parvum*, *P. minimum*, and *X. fastidiosa* would result in distinct changes in the microbiome and phenolic metabolites across different plant tissues in plants that were mostly asymptomatic. This was hypothesized because of their individualized lifestyles and infection mechanisms that include the production of species-specific virulence factors (i.e., cell wall-degrading enzymes and phytotoxins). Furthermore, by focusing on plants that did not yet have foliar symptoms, there would be reduced microbiome impact from the lack of photosynthate production and associated changes in host physiology. Currently, a systematic study comparing the impact of both fungal and bacterial pathogens on the microbiome and secondary metabolites across different plant tissues has not been undertaken in grapevines. Nevertheless, multiple studies have examined one pathogen on grapevine host physiology (Wallis and Chen, 2012; Wallis et al., 2013, Wallis and Galarneau, 2020). Other studies have also examined the effects of one microbe (i.e., pathogen) on others as influenced by shifts in host physiology (Wallis et al., 2022). However, examination of the larger bacterial and fungal microbial communities in response to multiple pathogen infections in grapevine and linking them to host chemistry shifts has been lacking.

Therefore, in this study, we tested the impact of inoculating five distinct pathogens (four fungal and one bacterial) on host secondary metabolism and microbiome development across both above- and below-ground compartments of grapevines. As these pathogens, especially the fungi, persist as generally asymptomatic for many years (barring the development of lesions), the effect on the microbiome during their latent period was analyzed in this study. Thus, we attempted to demonstrate that the impact of pathogen infection on microbiome community structure is most acute at the site of inoculation and that different pathogens show different levels of colonization and penetrance within this tissue type. We hypothesized that specific non-focal bacterial and fungal species were differentially enriched or depleted following inoculation with each pathogen. We then attempted to show that these groups were largely non-overlapping. Finally, we explored the impact of pathogen inoculation on the abundance of selected phenolic metabolites and showed that treatment with different pathogens may result in identifiable signatures of altered metabolism. Collectively, the obtained data may help define the differences and commonalities in microbial and metabolic shifts that occur following exposure to five agronomically relevant disease-causing agents of grapevines.

## 2 Materials and methods

### 2.1 Plant materials and pathogen inoculation treatments

Grapevine varieties used in all experiments were 2-year-old Cabernet Sauvignon (French 337) grafted onto 101–14 MG rootstock obtained from a commercial nursery. Vines were kept in a greenhouse with climate-controlled conditions (temperatures ranging/averaging between 20 °C and 30 °C, humidity averaging 25%, and auxiliary lighting scheduled to maintain daylight conditions for 14 hours). All vines chosen for this experiment had at least two scion branches. Greenhouse experiments were conducted in the summer of 2022.

Grapevines were arranged in a completely randomized block experimental design using two spatial blocks. Ten plants each received no treatment (as a negative control), mock inoculation, or inoculation by one of the five different pathogens: *D. seriata*, *E. lata*, *N. parvum*, *P. minimum*, or *X. fastidiosa*. The fungal pathogens were obtained from Dr. Kendra Baumgartner, USDA-ARS, Davis, CA. These were isolations originally made from California, USA, from woody host plants (Wallis et al., 2022). The *X. fastidiosa* ssp. *fastidiosa* strain was the SL strain isolated from a grapevine in California (Wallis and Chen, 2012).

Pathogens were inoculated directly into the stems of vines (10 replicate plants per treatment group). To account for potential shifts resulting from the wounding process that occurred during inoculation, a second control group was included (in addition to a no-treatment control), termed “wounded”, which included a mock inoculation with potato dextrose broth following inoculation. The fungal trunk pathogen inoculations and mock inoculation consisted of creating a 10-mm wound in the bark of the first internode of the scion (middle of node) above the scion-rootstock grafting point with a 10-mm cork borer. Following the wounds, the mock inoculations had an 8-mm potato dextrose agar (PDA; BD Scientific, Franklin Lakes, NJ, USA) plug added, whereas for the fungal pathogens, an 8-mm mycelium-colonized agar plug was added. The plugs were applied mycelium-side down and wrapped in parafilm to allow for localized colonization to occur without contamination. For *X. fastidiosa* inoculation, a sterile liquid suspension of *X. fastidiosa* was created from loop swabs of cultures grown on periwinkle wilt agar (2 weeks old) into a sterile 2-mL sample vial containing 500 µL of DNA-free sterile water (concentration of roughly 1 × 10^5^ CFU/mL) (Wallis and Chen, 2012). The suspension was introduced into grapevines by creating five wounds with a 16-gauge needle (BD Scientific) throughout the second to third internodes of the scion above the grafting site. Five micro-droplets (approximately 10 µL each) were dispensed using a 1-mL Sub-Q syringe with a 26-gauge needle (BD Scientific) to the wounds, allowing the bacteria to enter the grapevine vascular system via capillary action (Wallis et al., 2013).

After 16 weeks, the plants were first assessed for disease (i.e., stem lesion development) and then harvested. For all plants, roots, inoculation points, leaves, and the stem above the inoculation point were harvested, with tissues placed into a 15-mL tube. These sample tubes were immediately flash frozen in liquid nitrogen and stored in −80 °C for further analysis. Root sampling involved collecting roughly 25 g of tissue and consisted of a randomly collected mixture of tap and feeder roots. The inoculation site sample involved collecting the entire internode where the fungal or bacterial inoculations occurred, or the equivalent site for the non-wounded and mock-inoculation controls. The distal stem samples involved collecting the last fully expanded internode from a different branch than the one that was inoculated, roughly 50 cm from the inoculation site on the different branch. The leaf samples involved collecting five leaves (roughly 25 g) randomly from the canopy of the grapevines.

To assist in determining fungal pathogen inoculation success, prior to processing the inoculation site samples for chemistry and DNA extraction, the intact internodes were photographed next to a ruler to measure the linear size of the developing cankers (Wallis et al., 2013).

### 2.2 Grapevine sample preparation and DNA extraction

For all tissues, samples were pulverized while submerged in liquid nitrogen using a mortar and pestle, with two aliquots of 0.10 g made into separate tubes (Wallis and Chen, 2012). These were kept at −20 °C until further extractions. One of these aliquots was saved for phenolic compound extraction as described below. The other aliquot was used for DNA extraction. Genomic DNA extraction consisted of using the Plant Mini Kit from Macherey-Nagel (Bethlehem, PA, USA) according to the manufacturer’s recommendations. For the non-wounded controls and *X. fastidiosa*-inoculated samples, an aliquot of the extracted DNA was used to determine *X. fastidiosa* titer by qPCR to verify inoculation success (Wallis et al., 2013).

### 2.3 Library preparation and sequencing

Dual-indexed primers for Illumina sequencing were used to amplify the V3–V4 region of the 16S rRNA gene (341F: 5′-CCTACGGGNBGCASCAG-3′ and 785R: 5′-GACTACNVGGGTATCTAATCC-3′) (Herlemann et al., 2011) or the ITS2 region of fungal rRNA (ITS9F: 5′-GAACGCAGCRAAIIGYGA-3′ and ITS4R: 5′-TCCTCCGCTTATTGATATGC-3′) (White et al., 1990; Menkis et al., 2012). Both of these used 10 ng of template DNA and Invitrogen Platinum Hot Start PCR Master Mix (2X, Catalog No. 13000014, Waltham, MA, USA) along with 0.4 μg bovine serum albumin and host plastid and mitochondrial clamps for the 16S library (0.75 μM final each, PNA Bio., Thousand Oaks, CA, USA). PCRs were performed in triplicate with the following settings: initial denaturing consisting of heating to 98 °C for 180 s; followed by 30 cycles of 98 °C for 45 s for denaturing, 78 °C for 10 s for a transition to the annealing step, 55 °C for 60 s for annealing, and 72 °C for 90 s for elongation; and a final extension of 72°C for 10 min. PCR product was quantified using a Qubit 3.0 fluorometer (Invitrogen, Waltham, MA, USA) and Qubit 1X dsDNA High Sensitivity Assay Kit (Cat. No. Q33266, Invitrogen). The PCR product (100 ng) for each sample was pooled. Next, 1 μg of the pool was cleaned using Agencourt AMPure XP beads (Beckman Coulter, West Sacramento, CA, USA), as follows. To each sample, beads were added at a 1.5× volume rate. The samples were then washed twice with 70% ethanol (with a volume of ethanol that was 200 μL greater than the volume of the existing sample). Lastly, the samples were resuspended in 30 μL of sterile nuclease-free water. The final pooled sample was quantified with a Qubit 3.0 fluorometer (Invitrogen, Waltham, MA, USA) and Qubit 1X dsDNA High Sensitivity Assay Kit (Cat. No. Q33266, Invitrogen). The final concentration was adjusted to 10 nM for sequencing at the University of California, Berkeley, Vincent J. Coates Genomics Sequencing Laboratory using an Illumina MiSeq platform with kit v3 (Illumina, San Diego, CA, USA) to generate 300PE reads.

Raw reads were demultiplexed using QIIME2 (Bolyen et al., 2019) and processed using DADA2 (Callahan et al., 2016) to create amplicon sequence variants (ASVs). Bacterial taxonomies were assigned using the SILVA SSU database 138 (released on December 16, 2019) of the 16S rRNA V3–V4 gene regions as a classifier trained in QIIME2 (Bolyen et al., 2019). Fungal taxonomies were assigned using the UNITE database v9.0. ASV tables were imported into R (v4.2.3). A total of 10.86 and 9.73 million reads for 16S and ITS amplicons, respectively, were obtained. Samples with fewer than 1,000 clean reads were discarded. This resulted in 29,266 and 15,381 ASVs for 16S and ITS datasets, respectively, before agglomerating ASVs by genus. The R package phyloseq v1.40.0 (McMurdie and Holmes, 2013) was used to determine the alpha and beta diversity. For the alpha diversity (genus level), the number of reads per sample was rarefied to even depth, and the diversity measure “Shannon” was used. Tukey’s Honestly Significant Difference (HSD) test was used to detect significance due to fungal pathogen inoculation treatments. The beta diversity was analyzed via principal coordinates analysis (PCoA) using the Bray–Curtis dissimilarity distance, and a constrained analysis of principal coordinates on the Bray–Curtis distances was performed with the variable “Treatment” to constrain the ordination. Differences between sample groups were determined using a Permutational Multivariate Analysis of Variance (PERMANOVA). Differentially abundant taxa at the genus level were determined using the R package DESeq2 v1.36.0 (Love et al., 2014), which uses a negative binomial generalized linear model and performs a Wald test. Prior to analysis, a pseudocount of 1 was added to the ASV count table. The R package “indicspecies” v1.8.0 was used for the indicator species analysis (De Cáceres and Legendre, 2009).

### 2.4 Phenolic compound analyses

Using one of the 0.10 g pulverized tissue aliquots, phenolics were extracted in 0.5 mL of 100% methanol (Fisher Scientific, Pittsburgh, PA, USA) overnight at 4 °C. The extract was removed, and the remaining pellet was re-extracted in another 0.5 mL of methanol overnight at 4 °C. Both supernatants were combined to yield 1 mL total methanol extract. High-performance liquid chromatography (HPLC) was then performed to examine phenolics according to the method and conditions of Wallis et al. (2013). Phenolic compounds were analyzed on a Shimadzu (Columbia, MD, USA) LC-20AD HPLC system equipped with an Ascentis RP C18 column (Sigma-Aldrich, St. Louis, MO, USA), connected to a Shimadzu PDA-20AD photodiode array detector, with a binary gradient proceeding from 95% water to 100% methanol (both with 0.2% acetic acid added) and back to starting conditions over 40 min. Compound peaks were identified by matching the retention times and UV/Vis maxima with those of commercial standards obtained from Millipore-Sigma (St. Louis, MO, USA) (Wallis et al., 2013). Based on these identifications, peak areas were converted to gram amounts per gram fresh weight of tissue by running standard curves of reference standards within the same compound subclass: catechin for flavan-3-ols, procyanidin B2 for procyanidins, quercetin glucoside for flavonoid glycosides, or resveratrol for stilbenoids (Wallis and Chen, 2012).

For all statistical analyses to assess differences in phenolic compounds, JMP (SAS Institute, Cary, NC, USA) version 16 was utilized with α = 0.05. Multivariate analyses of variance (MANOVAs), with Pillai’s trace test statistic, and follow-up analyses of variance (ANOVAs), were used to determine differences in phenolic compound levels due to inoculation treatments in each tissue sample separately. Follow-up Tukey’s HSD multiple comparison tests were performed when appropriate. Blocks were included in each ANOVA but removed when not significant, and no interactions with blocks and the other main effects were observed.

Furthermore, canonical discriminant analyses were performed to observe whether the inclusion of certain phenolic compounds could separate different inoculation treatments. This was performed in a stepwise manner with a linear model and included compounds when *p* < 0.05 but excluded compounds if *p* > 0.05. The accuracy of the discriminant analyses was derived accordingly.

## 3 Results

In this study, the impact of four fungal pathogens (*D. seriata*, *E. lata*, *N. parvum*, and *P. minimum*) and one bacterial pathogen (*X. fastidiosa*) on the host metabolism and microbiome development of 2-year-old grapevines was tested. After 16 weeks post-inoculation and growth in a greenhouse, the inoculation sites of plants treated with a fungal pathogen were surveyed to determine the lesion length (canker). The fungi *E. lata* and *N. parvum* resulted in significantly longer lesion length compared to the wounded control, indicating successful inoculations and symptom development for these pathogens (**Supplementary Figure 1**). *X. fastidiosa* inoculations did not show any canker lesions because of the less invasive pin-prick inoculation process commonly used in *X. fastidiosa* studies. Additionally, this bacterial pathogen does not cause canker-like lesions on shoots, whether it is inoculated artificially or naturally by its insect vectors. There were no observed foliar symptoms due to infection by any of the pathogens. Additional confirmation of infections was made and confirmed by replating the pathogens and identification by consistent morphology or, for *X. fastidiosa*, positive qPCR results.

**Figure 1:**
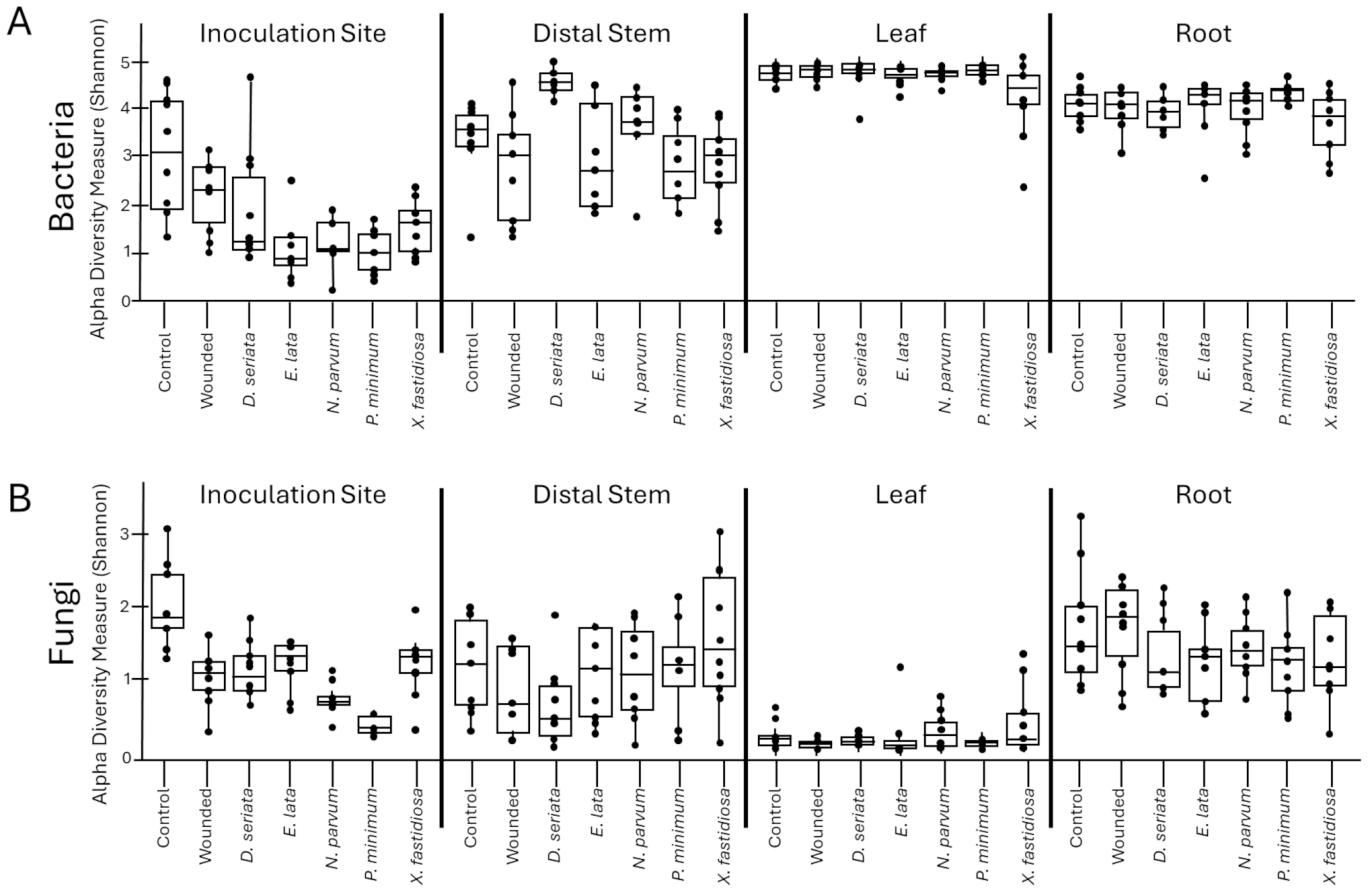
Alpha diversity analysis (Shannon’s index) for **(A)** bacterial and **(B)** fungal communities.

Analysis of the alpha diversity of bacterial and fungal microbiome datasets across treatments and sample types revealed several patterns (**Figure 1**; **Supplementary Figure 2**). First, bacterial diversity at the inoculation site was significantly lower than that of every other sample type for all the pathogen-treated groups (*p* adj. < 0.01), and diversity at the inoculation sites for pathogen-impacted plants was lower than that of the unwounded control (*p* adj. < 0.05). Other significant differences were found for the inoculation site samples of *P. minimum* and the wounded control (*p* adj. < 0.01) and distal stems of *D. seriata* and wounded vines (*p* adj. < 0.001). Second, fungal alpha diversity appeared overall to vary by sample type, with leaf samples trending with a lower alpha diversity compared to other sample types. As was observed for the bacterial dataset, wounding and pathogen at the inoculation site generally led to lower alpha diversity compared to the unwounded control, and significant differences were observed in all treatments with the exception of *E. lata* and *X. fastidiosa* samples (*p* adj. < 0.01). Across both datasets, *P. minimum*-treated plants showed the lowest mean diversity at the site of inoculation (mean Shannon index of 1.01). Collectively, these data suggest that differences in alpha diversity were observable across treatments, with pathogen inoculation and the wounding process generally leading to a reduction in diversity levels, specifically at the site of inoculation (**Figure 1**).

To assess the influence of pathogen inoculation on the beta diversity, we analyzed community structure through PCoA using the Bray–Curtis distance for all samples within the bacterial and fungal datasets (**Supplementary Figures 2**, **3**). The data demonstrate significant separation of samples according to sample type (PERMANOVA, *r*^2^ = 0.412 and 0.574; *p* < 0.001) for both bacterial and fungal communities, but significant separation according to pathogen treatment occurring within the inoculation site, distal stems, and leaves for the bacterial dataset (PERMANOVA, *r*^2^ = 0.485, 0.490, and 0.251; *p* < 0.001) and only within the inoculation site for the fungal dataset (PERMANOVA, *r*^2^ = 0.557; *p* < 0.001) (**Supplementary Figures 2**, **3**). This ordination data from the inoculation site also revealed clear clustering of specific treatment groups within the bacterial 16S rRNA dataset for fungal pathogen treatments, suggesting that the application of one type of pathogen may strongly influence the composition of the microbiome belonging to the other microbial kingdom. Infection by fungal pathogens always significantly impacted the fungal and bacterial communities of inoculation sites (PERMANOVA, *D. seriata*, *r*^2^ = 0.143 and 0.137; *E. lata*, *r*^2^ = 0.226 and 0.288; *N. parvum*, *r*^2^ = 0.460 and 0.185; *P. minimum*, *r*^2^ = 0.484 and 0.427; *p* < 0.05), while infection by *X. fastidiosa* did not but rather had a significant effect on the fungal and bacterial microbiomes of leaf and distal stem samples (PERMANOVA, leaf, *r*^2^ = 0.170 and 0.409; distal stem, *r*^2^ = 0.193 and 0.169; *p* < 0.05).

**Figure 2:**
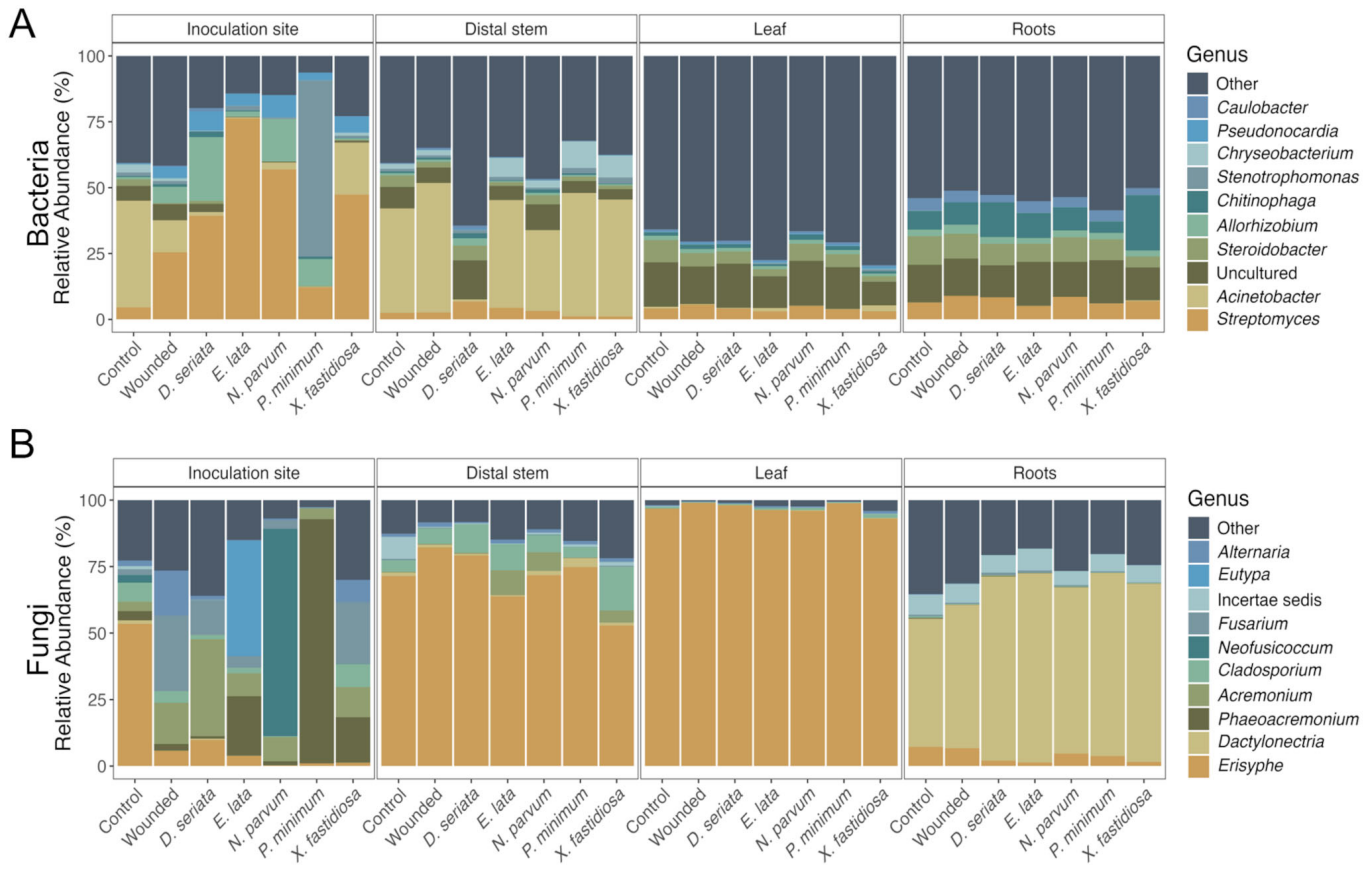
Relative abundance plots for the **(A)** bacterial and **(B)** fungal microbial communities for inoculation site, distal stem, leaf, and root samples.

Additionally, relative abundance plots of bacterial and fungal genera indicated that pathogen infection led to significant changes (*p* < 0.05) of the microbiome, primarily in the inoculation site compared to the wounded control (**Figures 2A, B**, respectively). Infection with the fungus *P. minimum* resulted in a large increase of *Stenotrophomonas* spp. within the bacterial community of the inoculation site (**Figure 2A**; **Supplementary Figure 4**). Additionally, a *D. seriata* infection led to a large decrease in the *Acinetobacter* genus in distal stems (**Figure 2A**; **Supplementary Figure 4**). *Eutypa*, *Neofusicoccum*, and *Phaeoacremonium* were among the top 10 most abundant genera within their respective inoculation site samples, indicating that they were highly successful at colonizing these tissues (**Figure 2B**; **Supplementary Figure 5**). Specifically, the majority of inoculation site samples treated with *E. lata*, *N. parvum*, and *P. minimum* showed dominance (relative abundance represents greater than or equal to 50% of the total) of their respective genera, while inoculation site samples treated with *D. seriata* and *X. fastidiosa* led to an average relative abundance of 19.29% and 6.66% of their respective genera (**Supplementary Figures 4**, **5**). These results also suggest successful inoculation and infection by all five pathogens.

Some of the applied pathogens (e.g., *P. minimum*) are specific species or strains of otherwise cosmopolitan fungal genera, which can be found in a wide variety of environments, including the growth spaces used for this experiment, potentially confounding our ability to detect enrichment of our pathogens within and across treatments. For example, a number of ASVs within our 16S rRNA dataset were assigned to the genus *Phaeoacremonium*, and reads assigned to this genus were observed in a range of samples, including but not limited to those inoculated with this pathogen (**Supplementary Figure 6**). Fortunately, an ASV-level analysis of all *Phaeoacremonium* ASV abundances across the dataset revealed that ASVs belonging to the pathogen and environmentally derived strains were largely distinguishable across inoculation site samples, with clear, non-overlapping patterns in their abundance patterns across treatment groups.

**Figure 3:**
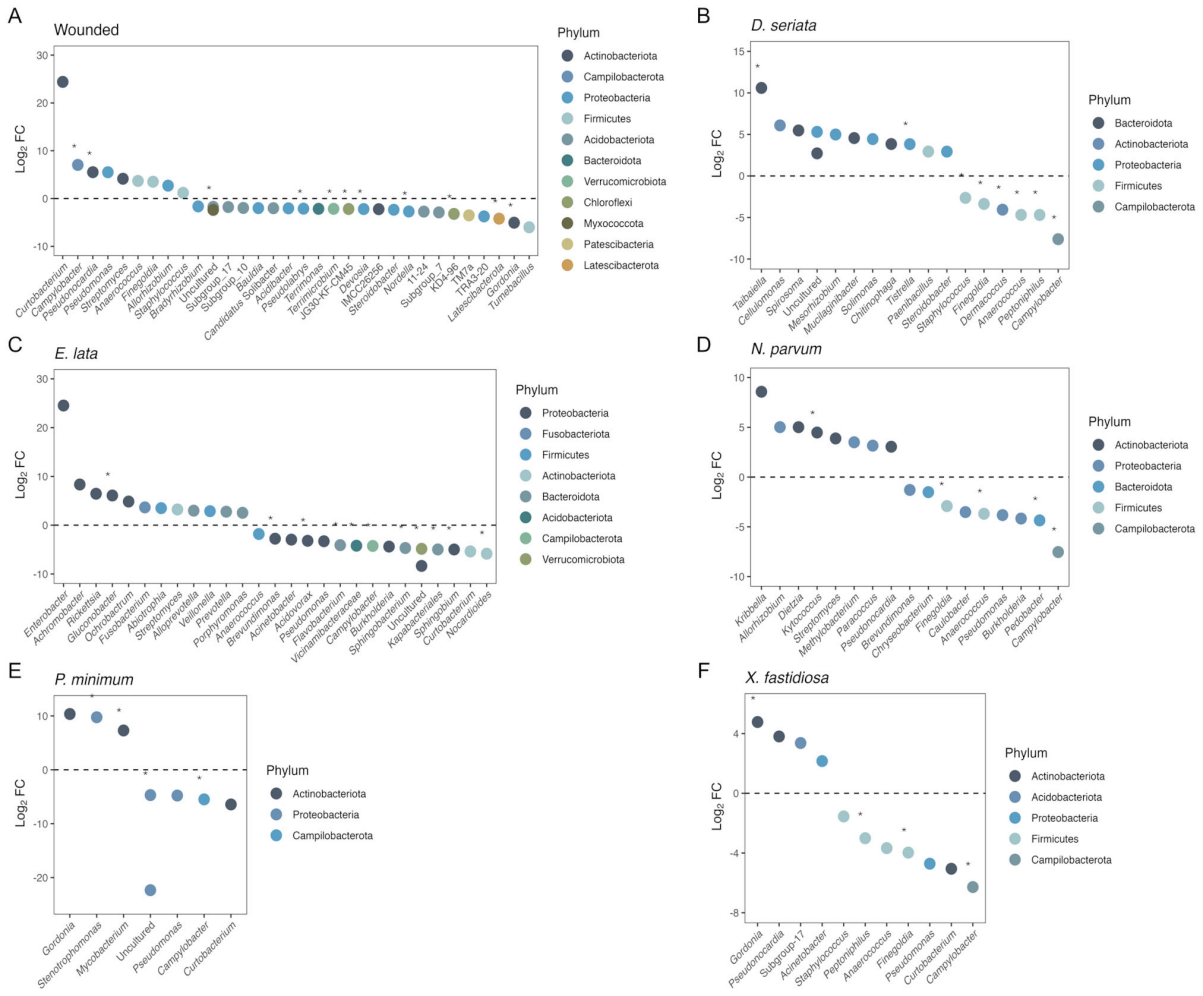
Bacterial differential genera for inoculation site **(A)** wounded, **(B)** *Diplodia seriata*, **(C)** *Eutypa lata*, **(D)** *Neofusicoccum parvum*, **(E)** *Phaeoacremonium minimum*, and **(F)** *Xylella fastidiosa* samples. Asterisk (*) indicates significantly enriched or depleted genera determined via DESeq2 and indicator species analyses.

**Figure 4:**
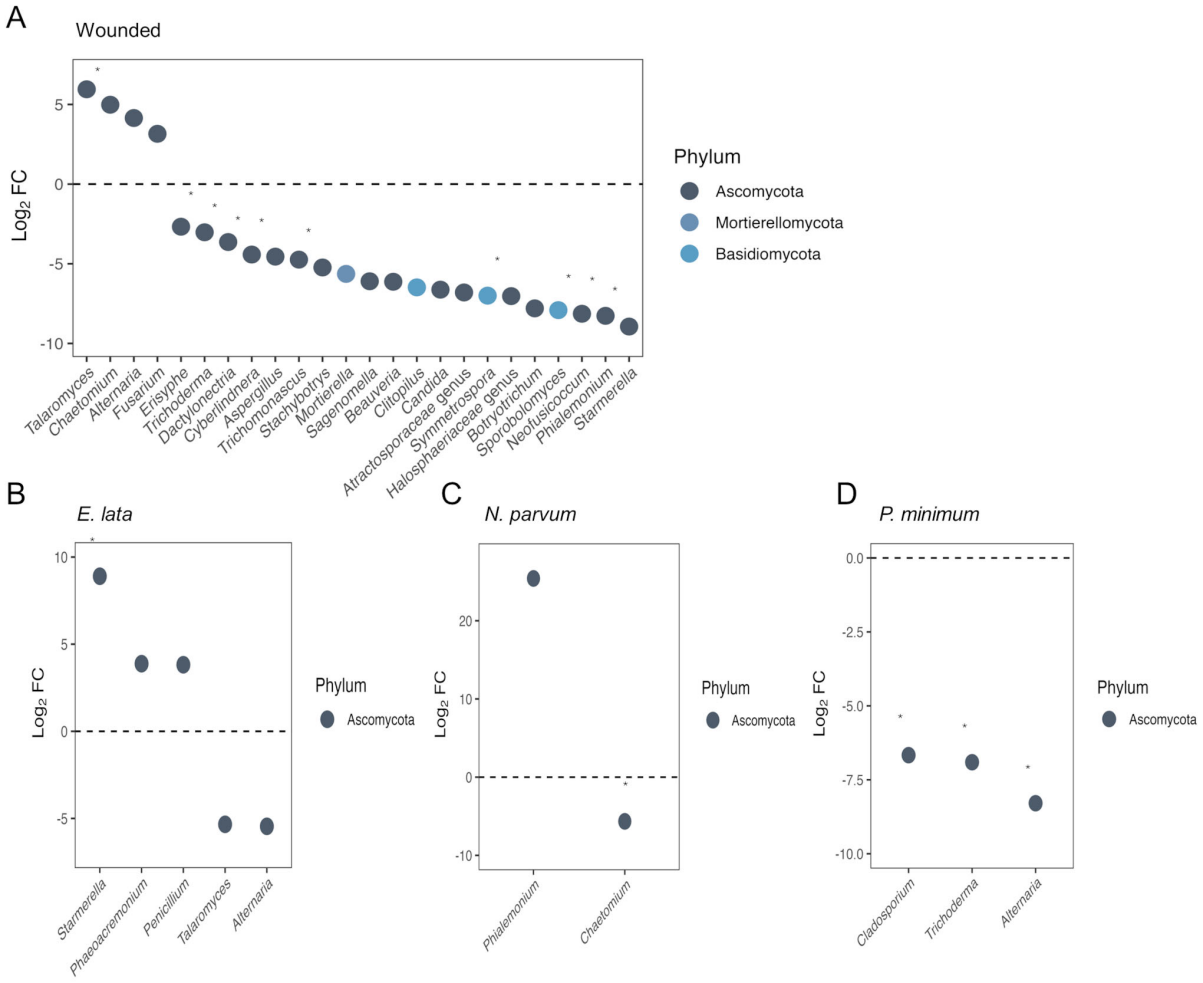
Fungal differential genera for inoculation site **(A)** wounded, **(B)** *Eutypa lata*, **(C)** *Neofusicoccum parvum*, and **(D)** *Phaeoacremonium minimum* samples. Note that there were no fungal differential genera for *Diplodia seriata* or X. fastidiosa. Asterisk (*) indicates significantly enriched or depleted genera determined via DESeq2 and indicator species analyses.

Interestingly, these relative abundance analyses revealed clear enrichment of microbial lineages in addition to the inoculated pathogen within specific treatment groups. As an example, the bacterial dataset revealed the aforementioned clear enrichment of the bacterial genus *Stenotrophomonas* within inoculation site samples treated with the fungal pathogen *P. minimum* (**Supplementary Figure 4**). To help identify both bacterial and fungal taxa significantly enriched by inoculation with each of our pathogen groups, we performed both DESeq2 and indicator species analyses across our two datasets in inoculation site samples and determined genera as differential if candidates were found either enriched or depleted in both analyses (*p* adj. < 0.05 for DESeq2 and *p* < 0.01 for indicator species). For the bacterial dataset, every pathogen treatment, as well as the wounded control, led to significant enrichment and/or depletion of at least four bacterial taxa, with the wounded control and *E. lata* infections causing the most changes (a total of 11 differential genera) (**Figure 3**; **Supplementary Figure 7**). The majority of the differential genera across treatments belonged to the phyla Actinobacteriota, Firmicutes, Bacteroidota, and Proteobacteria. Notably, the genus *Campylobacter* was significantly depleted in every pathogen treatment but enriched in the wounded control. The number of differential fungal taxa was much lower than that of the bacterial taxa; only the wounded control, *E. lata*, *N. parvum*, and *P. minimum* infections significantly affected fungal genera (**Figure 4**; **Supplementary Figure 8**). These taxa were found mainly within the class Sordariomycetes and phylum Ascomycota.

**Figure 5:**
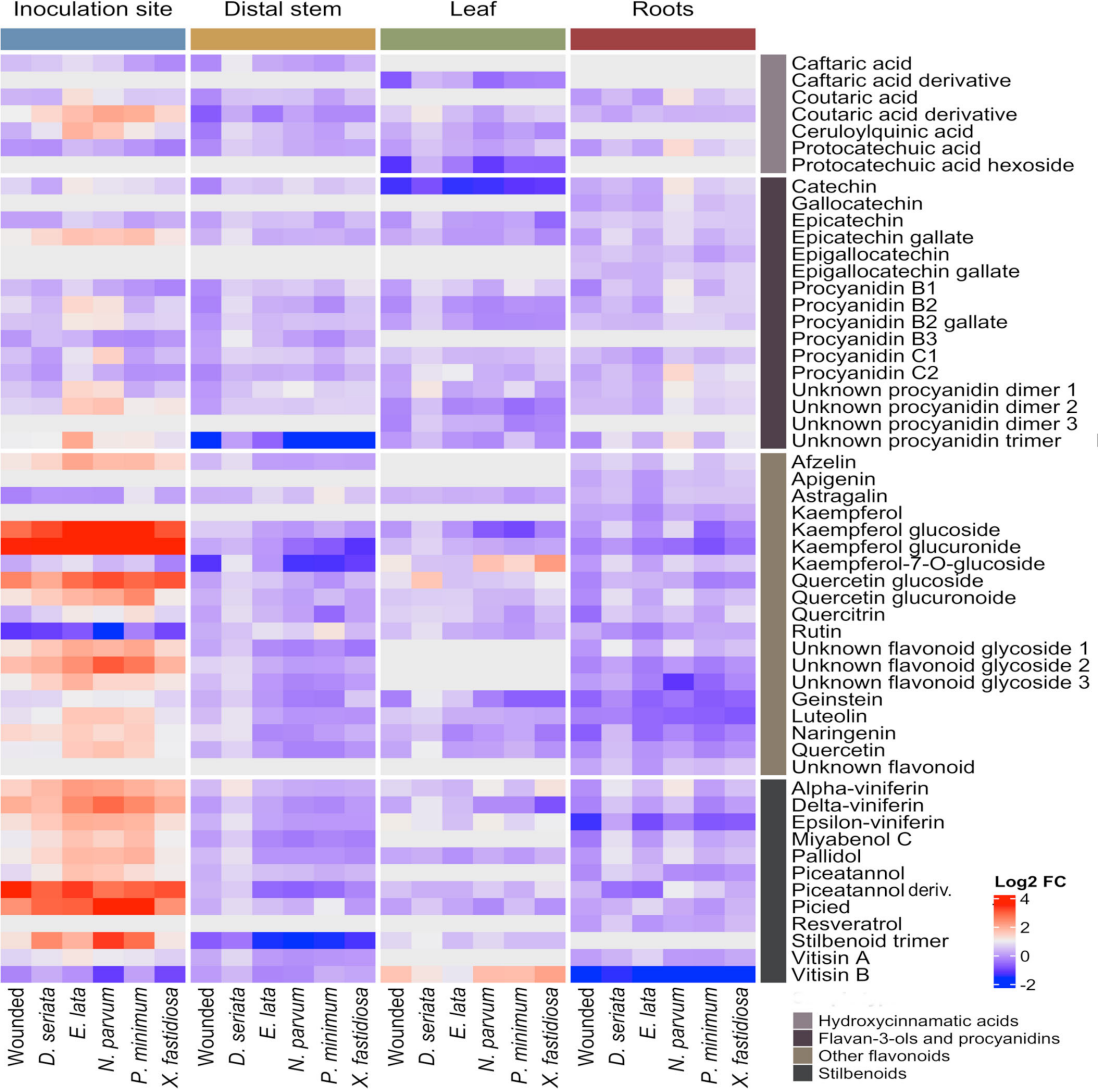
Pathogen inoculation led to unique phenolic compound levels across all four types of grapevine tissue. Heatmap summarizes log2 fold change (log2 FC) of phenolic compounds (mg/g fresh weight tissue) for all pathogen treatments and different grapevine tissues. Cells colored light gray indicate that the corresponding compound was below the detection limit in the examined tissue.

To better understand the impact of these prominent grape pathogens on their host, we analyzed the abundances of phenolic secondary metabolites, which are indicators of defense responses. Similar to our 16S and ITS analysis results, we observed the greatest changes in phenolic metabolites in the inoculation site tissues. Around the inoculation site, most of the analyzed compounds were significantly greater within pathogen-infected plants than controls (Pillai’s trace = 4.964; *F*_6,57_ = 2.228; *p* < 0.001) (**Figure 5**; **Supplementary Data 1**). A total of 25 compounds analyzed had greater levels around *E. lata* or *N. parvum* lesions than at least one of the controls, with stilbenoid compound levels generally having the greatest differences. Away from the inoculation site, stem tissues had compound levels significantly increased when plants were infected (Pillai’s trace = 5.237; *F*_6,50_ = 2.076; *p* < 0.001) (**Figure 5**; **Supplementary Data 1**). However, for a total of 17 compounds, plants infected with *D. seriata* had greater levels than at least one of the other treatments, especially stilbenoid levels. Leaf tissues similarly had greater phenolic levels in infected plants compared to healthy controls (Pillai’s trace = 4.618; *F*_6,59_ = 2.692; *p* < 0.001) (**Figure 5**; **Supplementary Data 1**). *D. seriata* had greater levels of nine examined phenolic compounds compared to other treatments, and *X. fastidiosa* had greater levels of four phenolic compounds. Root tissues did not appear to have different phenolic levels among treatments (Pillai’s trace = 5.564; *F*_6,50_ = 0.555; *p* = 0.953) (**Figure 5**; **Supplementary Data 1**). Follow-up ANOVAs on individual compounds were performed, with significance observed only for miyabenol C and a piceatannol derivative, with the latter having greater levels in *D. seriata*-infected plants than other treatments.

**Figure 6:**
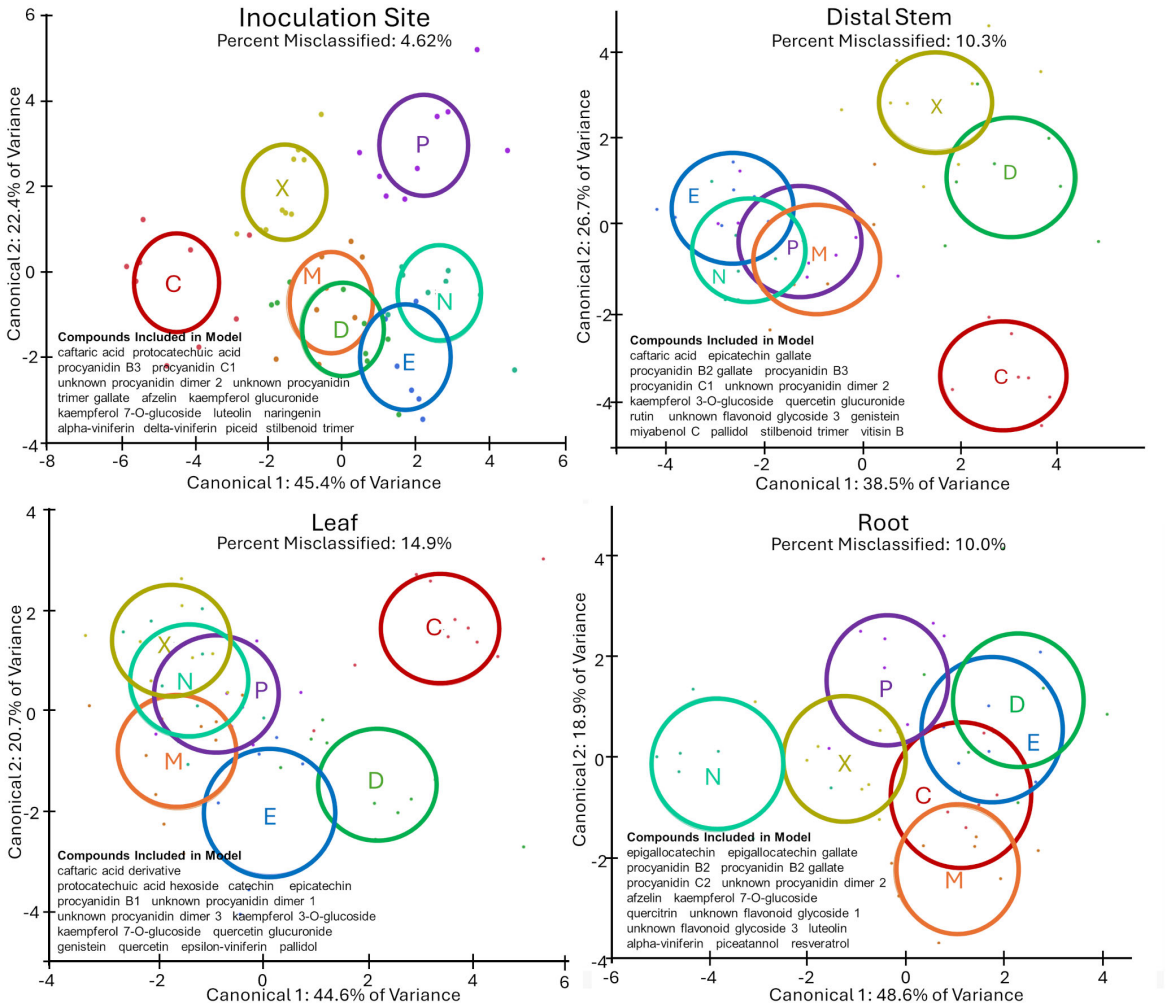
Canonical discriminant analysis plot using a stepwise linear-discriminant method to separate treatments according to phenolic profiles, with compounds included in the model provided. C, non-infected control; M, mock-inoculated control; D, *Diplodia seriata* infected; N, *Neofusicoccum parvum* infected; E, *Eutypa lata* infected; P, *Phaeoacremonium minimum* infected; X, *Xylella fastidiosa* infected.

Discriminant analyses using a stepwise linear method were successful at separating the infection status of plants based on phenolic profiles in all tissues. At the inoculation sites, the discriminant analyses produced a model with 15 compounds, which covered over 67.8% variability in the first two canonicals, with 95.4% correctly identified (**Figure 6**). Although profiles from plants infected with *P. minimum* overlapped with mock-inoculated plants, most treatments were well-separated by the model.

The discriminant analysis model for phenolic profiles in stem tissue away from the distal site, which had 15 compounds in the model and explained 65.2% of the overall variability, had 89.7% of the samples correctly identified (**Figure 6**). Non-wounded controls and plants infected with either *D. seriata* or *X. fastidiosa* were better separated than those receiving the other treatments.

Phenolic profiles of leaves did not have as successful separation due to discriminant analysis, with 85.1% of samples correctly identified when 14 compounds were used, and the model had the first canonical variable explain 65.2% of variance (**Figure 6**). Control samples and samples from plants infected with *D. seriata* had good separation due to phenolic profiles.

Discriminant analyses of root phenolic profiles explained 67.5% variance with the first two canonicals, correctly identified 90.0% of samples, and used 15 compounds (**Figure 6**). *N. parvum*-infected plants appeared to be well-separated from the other samples, which overlapped with each other.

## 4 Discussion

In this study, we conducted a systematic study comparing the impact of fungal and bacterial pathogens on the grape microbiome and phenolic metabolites across different plant compartments. We demonstrated that infection of both pathogen groups results in significant restructuring of the microbiome, including generalized reduction in alpha diversity and shifts in overall community structure at the site of infection. Other studies have demonstrated that pathogen infection can significantly alter the composition and functional dynamics of the plant-associated microbiome, often resulting in microbial dysbiosis (Chen et al., 2020). Infection by phytopathogens such as *Pseudomonas syringae* and *Fusarium oxysporum* can disrupt the equilibrium between beneficial and opportunistic microbial taxa in both the rhizosphere and phyllosphere, leading to decreased microbial diversity and shifts in community structure (Berg et al., 2020; Trivedi et al., 2020). Pathogen-induced modulation of plant immune responses and exudate profiles can further shape the microbial assembly, reinforcing a feedback loop between host physiology and microbiome composition (Berendsen et al., 2012). In grapevines, there is recent evidence both for and against this type of restructuring. For instance, infection by *Plasmopara viticola*, the causal agent of downy mildew, leads to cultivar-dependent shifts in rhizosphere bacterial communities (Duret et al., 2025). By comparing susceptible (Chardonnay) and resistant (Voltis) cultivars, Duret et al. (2025) found that infection reduced bacterial diversity in Chardonnay, notably decreasing the abundance of beneficial taxa such as *Pseudomonas*, *Sphingobacterium*, and *Rhizobium*, while increasing the prevalence of potentially pathogenic taxa like *Agrobacterium rhizogenes*. In contrast, the bacterial microbiome of Voltis remained relatively stable under infection pressure, suggesting a role for microbiome resilience in disease resistance. Together, these data demonstrate the need for host- and case-specific investigation of the impact of a pathogen on overall microbiome health.

Interestingly, we found that the majority of changes in microbiome diversity and structure were observed at the site of inoculation (stems), rather than systemically in other distal tissues (leaves and roots). This stands in contrast to some other recent studies from other systems. For example, *P. syringae* infection on *Arabidopsis* leaves leads to significant shifts in the root microbiome, suggesting that aboveground immune activation alters root exudation patterns and thereby influences microbial recruitment (Durán et al., 2018). Similarly, systemic acquired resistance (SAR) has been linked to changes in microbial assemblages in tissues distant from infection sites, potentially priming these regions for enhanced defense and altering their ecological niches (Yuan et al., 2018; Berens et al., 2019). These discrepancies between this study and prior studies may be in part explained by the fact that the grapevines used as hosts in the current study are long-lived and comparatively large, perennial plants, making distal shifts in the microbiome and the spread of pathogenic strains more unlikely to occur over short time frames (Wallis and Galarneau, 2020). These results also may differ from those of similar studies that allowed infections to progress to result in foliar symptoms to occur, as impacts on the health of leaves would likely result in cascading consequences on overall plant physiology and, in turn, the microbiome. Indeed, changes in the root microbiome were not observed in this study, and this was likely due to the lack of symptoms overall, with the exception of the lesions around the original inoculation sites for the fungal pathogens.

The experimental design of the present study also allowed us to compare the relative impact of five different pathogens on both the fungal and bacterial microbiomes. From these data, we found comparatively little evidence that the bacterial pathogen *X. fastidiosa* leads to alterations in fungal community structure; by contrast, we found strong evidence that fungal pathogens can significantly impact the bacterial microbiome of the host at the inoculation site and case-specific restructuring at other tissues. Several studies have shown that fungal infections, such as those caused by *F.oxysporum* or *Verticillium dahliae*, can reduce bacterial diversity and shift community composition, often enriching for opportunistic or pathogenic bacteria while depleting beneficial taxa like plant growth-promoting rhizobacteria (PGPRs) (Carrión et al., 2018; Mendes et al., 2023). These shifts may occur by causing tissue necrosis, which releases nutrients that support the growth of saprotrophic or commensal bacteria (Trivedi et al., 2020) but may also occur systemically through pathogen-mediated changes in the production of defense-related metabolites (Yuan et al., 2018). In grapevines, recent data suggest that microbial agents causing grapevine trunk diseases are associated with significant alterations in the endophytic bacterial microbiome. Asymptomatic vines tend to harbor higher abundances of beneficial genera like *Bacillus* and *Streptomyces*, which are known for their antifungal properties and may suppress pathogen colonization (Bekris et al., 2021). These bacteria are often depleted in symptomatic tissues, suggesting either that fungal infection directly suppresses beneficial bacteria or that disease conditions create a microenvironment less favorable for their persistence. Collectively, these studies highlight a dynamic and reciprocal relationship between fungal pathogens and the bacterial microbiome in grapevines, with implications for plant health and disease management.

Interestingly, certain bacterial taxa may also proliferate as secondary colonizers following fungal infection, forming polymicrobial communities that can exacerbate disease or, conversely, inhibit pathogen progression depending on context (Ginnan et al., 2020). In the present study, we demonstrated the strong enrichment of *Stenotrophomonas* following inoculation with the fungal pathogen *P. minimum*, while all three other fungal agents failed to induce an increase in this bacterial genus. Currently, there is no direct evidence of an interaction between *Stenotrophomonas* species and *Phaeoacremonium* fungi; however, *Stenotrophomonas* strains have been isolated from woody tissues of grapevines in a study of esca-related fungal diseases (Haidar et al., 2021). This study identified *Stenotrophomonas* strains from non-necrotic grapevine tissues that produced volatile organic compounds (VOCs) with significant antifungal activity against various pathogens, including those responsible for esca disease. The current study suggests a strong correlation between the presence of *Stenotrophomonas* and *P. minimum* in infected tissue, but additional studies will need to be conducted to indicate whether this relationship is antagonistic or synergistic in nature.

In terms of *P. minimum*, it appeared that this fungal species was enriched around the inoculation sites of *E. lata* and *X. fastidiosa*. This finding was surprising, as the other two pathogens (*D. seriata* and *N. parvum*) did not enrich this species. We hypothesized that *P. minimum* was able to take advantage of localized stress caused by infections. However, *D. seriata* and *N. parvum* may deploy antifungal metabolites to prevent colonization by other fungi, such as *P. minimum*, which prevented their enrichment.

*D. seriata* infection led to a significant depletion of the genus *Acinetobacter* in distal stems but not in the inoculation site. The molecular mechanisms and virulence factors by which *D. seriata*, the causal agent of *Botryosphaeria* dieback in grapevine, colonizes its host have yet to be described, and there are no reports of this pathogen’s behavior in microbe–microbe interactions. The distal stem samples in which we observed the *Acinetobacter* depletion were collected from a plant shoot separate from the shoot where the pathogen was applied (inoculation site), with the purpose of investigating systemic changes in the host. It may be hypothesized that pathogens could secrete proteins that travel throughout the vasculature of the plant, specifically the xylem, and this could negatively impact plant health and members of the native microbiome, such as *Acinetobacter*. Alternatively, a systemic response such as SAR could be the culprit of this significant change in the host microbiome. This strong immune response by the host has been reported to cause a change in the microbial community in tissues distant from infection sites (Berens et al., 2019; Yuan et al., 2018). Research into addressing these hypotheses is warranted.

Relative abundance plots showed fungal dominance by *Erysiphe* in distal stem and leaf samples and *Dactylonectria* in root samples. The *Erysiphe* genus contains a very common and pesky pathogen of grapevine, *Erysiphe necator*, which causes powdery mildew, and the genus *Dactylonectria* contains the soil-borne pathogen associated with black foot disease of grapevine. We suspect that our plant growing conditions led to the high presence of these additional pathogens in our samples. For example, powdery mildew spores already present in the greenhouse and/or starting material could have served as initial inoculum, and rootbound, stressed roots could have created a beneficial environment for soil-borne *Dactylonectria* to thrive in. Given these results, we cannot discard the possibility of pathogenic *Erysiphe* and *Dactylonectria* contribution to the shifts of the grape microbiome to some degree.

Finally, we were successful in identifying infection status using phenolic profiles, especially near the inoculation sites. This likely implies that the plant–microbe interactions result in a variety of unique physiological changes. Furthermore, mock inoculation caused different profiles than non-infected controls, implying that shifts associated with wound repair are detectable as well, even months after the wound was made. There was a notable increase in certain stilbenoid compounds, such as a stilbenoid trimer, around the inoculation sites of the fungal pathogens, but not for *X. fastidiosa*. Stilbenoids are notable defense-associated compounds, and the increase of these compounds suggests a robust attempt from the grapevine host to reduce the pathogen infections (Wallis and Galarneau, 2020). Perhaps the lower levels of stilbenoid trimers in *X. fastidiosa*-infected plants were due to grapevines not responding to the pathogen as a biotic threat but rather as an abiotic stress, which has been observed across many studies (Rapicavoli et al., 2018). Away from the inoculation site, especially in distal stem tissues and leaves, fewer separations were derived from the phenolic profiles. In general, *D. seriata* was the most separated along with non-infected controls. Indeed, these changes in phenolics could be hypothesized to greatly influence the bacterial microbial community structure, as discussed above. For its part, *X. fastidiosa* had distinct profiles in distal stem samples as well, but at that point, the bacteria likely spread into that tissue. This was in contrast to effects observed in the fungal microbial community composition, in which the distal samples were most affected when grapevines were infected with *E. lata*. One hypothesis is that the toxins produced by *E. lata* translocate to the distal tissue samples to affect the fungal microbial communities the most, instead of systemic induced changes in plant physiology from the infections.

Regardless, similar to observations made by Wallis et al. (2022), this study observed greater host-wide effects on phytochemistry due to *D. seriata* infections than *N. parvum* or *P. minimum* infections. The systemic changes in that case perhaps reduced the development of future fungal infections as well (Wallis et al., 2022). As for the root tissues, few significant changes were observed in phenolic levels. This likely resulted in poor separations among the different treatments using the discriminant analyses. However, plants infected with *N. parvum* appeared to have some separation from those that received the other treatments. It is possible that damage from that fungus impacted resource allocation to roots. In conclusion, these results revealed that phenolic profiling in different plant tissues could be linked to infection by different pathogens and that this is especially true at an active infection site. Thus, phenolic profiles could be utilized from around cankers to perhaps identify which pathogen infected the host plant as a complementary technique to those based on nucleic acid analyses, such as PCR and DNA sequencing.

## Data Availability

The datasets presented in this study can be found in online repositories. The names of the repository/repositories and accession number(s) can be found below: https:/github.com/dcolemanderr/Wallis-2025.
