# Supplementary material for: Different fungal and bacterial pathogen infections alter the grapevine microbiome and phenolic profiles in a localized and specific manner
Source: Front Plant Sci. 2026 Jun 16;17:1838241. doi: 10.3389/fpls.2026.1838241 (PMC13315004; doi:10.3389/fpls.2026.1838241)
Supplement: Supplementary file 1 [file Supplementaryfile1.pdf]

Supplementary Material

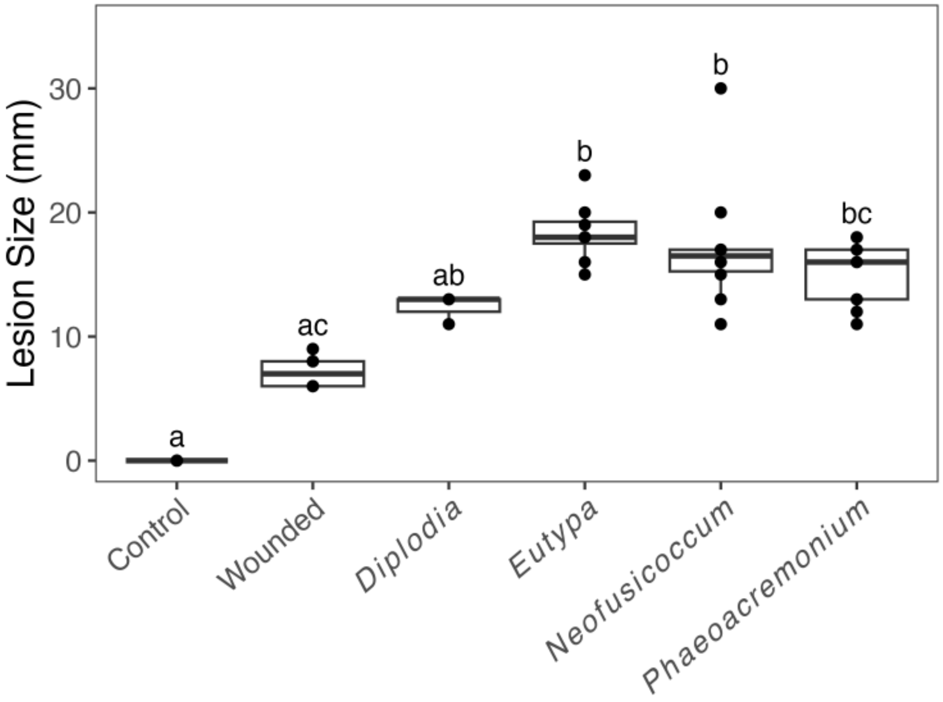

Supplementary Figure 1. Lesion lengths at the inoculation sites (or equivalent sites for controls). Significance was determined by Kruskal-Wallis test ($P < 0.0001$) followed by Dunn’s Kruskal-Wallis Multiple Comparisons test.

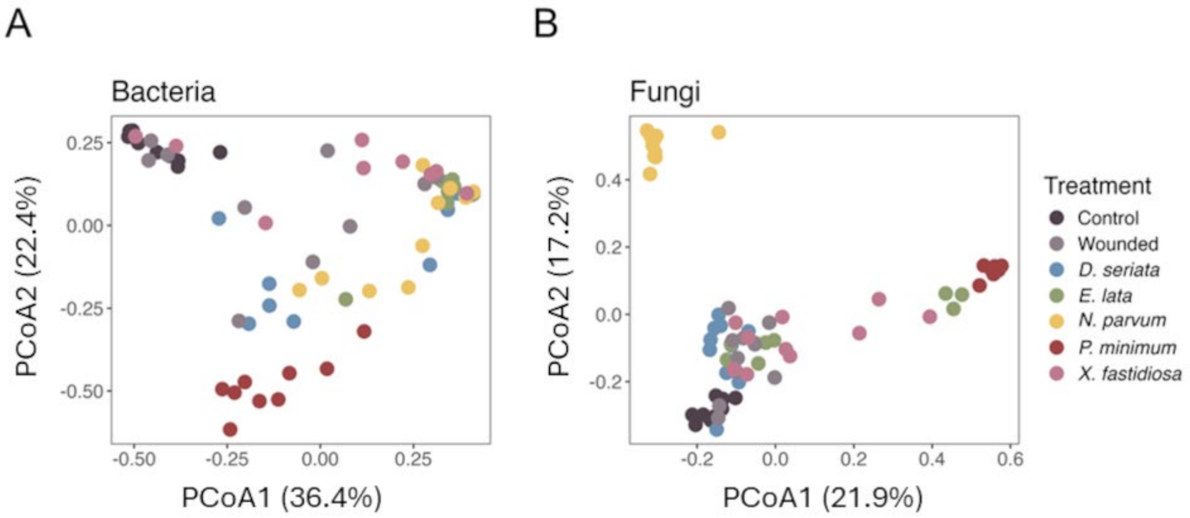

Supplementary Figure 2. Pathogen inoculation impacts fungal and bacterial community structure at the inoculation site. Beta diversity analysis for the (a) bacterial and (b) fungal microbial communities for inoculation site samples across all treatments (Permanova, P < 0.001).

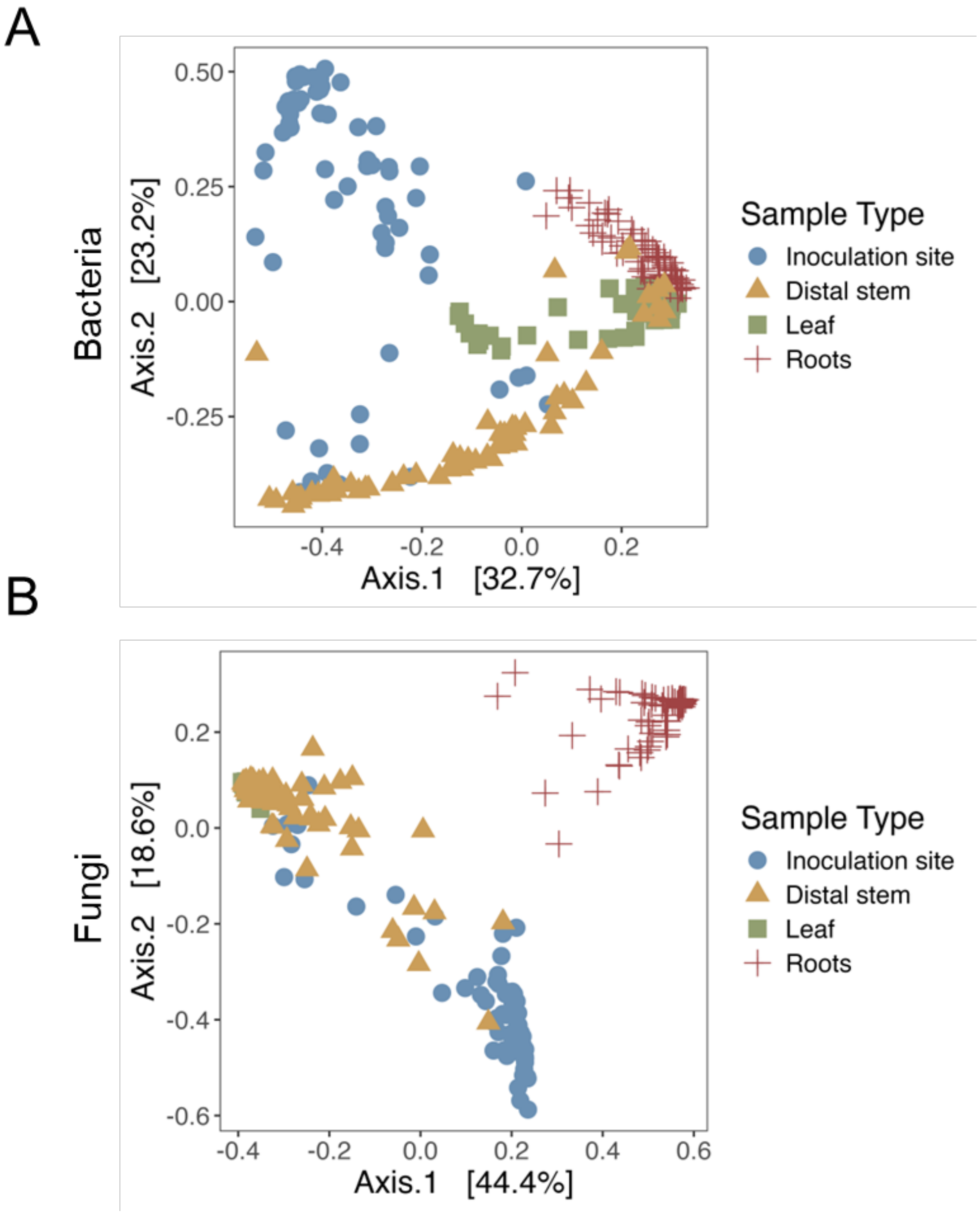

Supplementary Figure 3. Global (all samples) beta diversity analysis of A) bacterial and B) fungal communities.

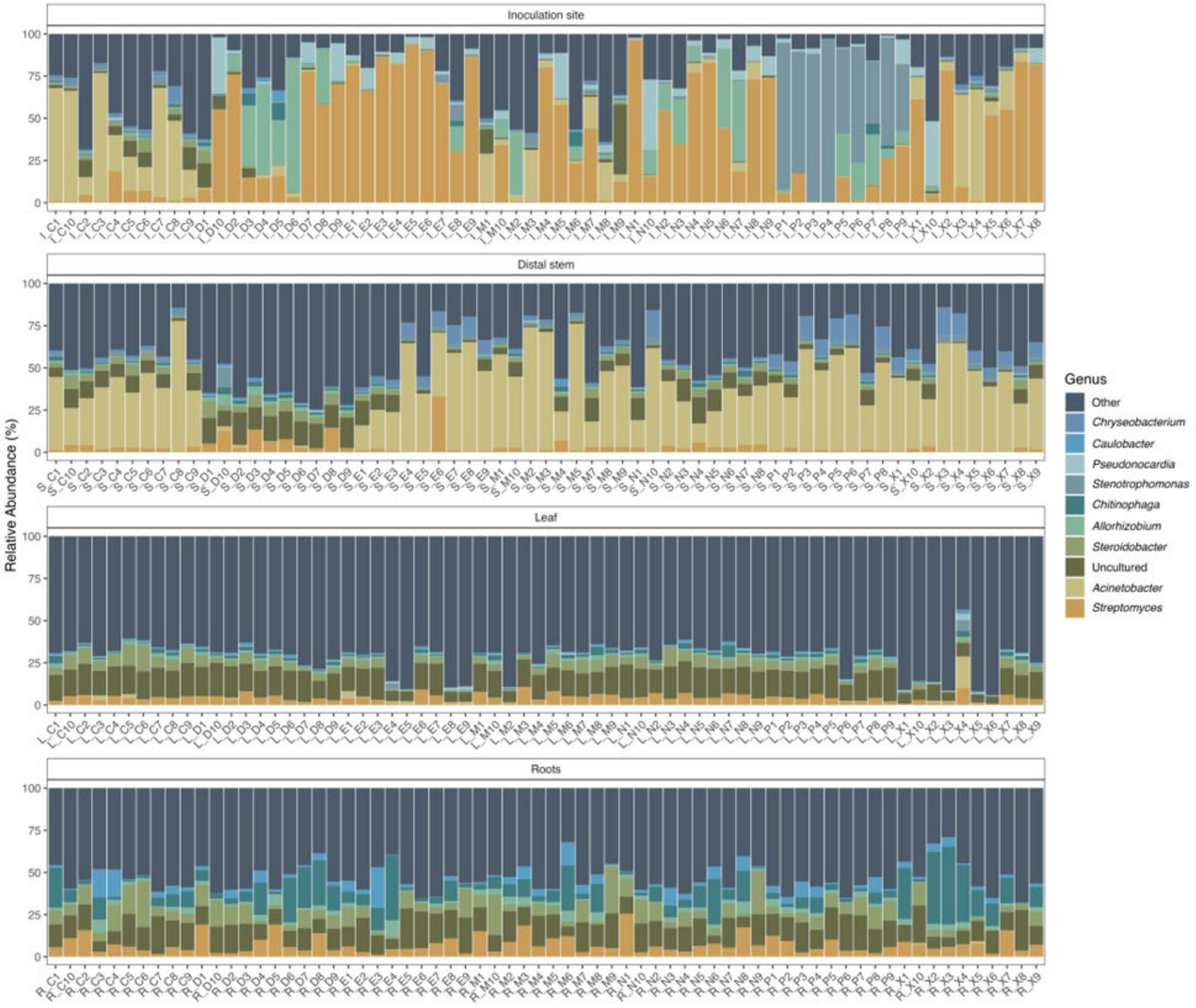

Supplementary Figure 4. Bacterial relative abundance plots for all individual samples.

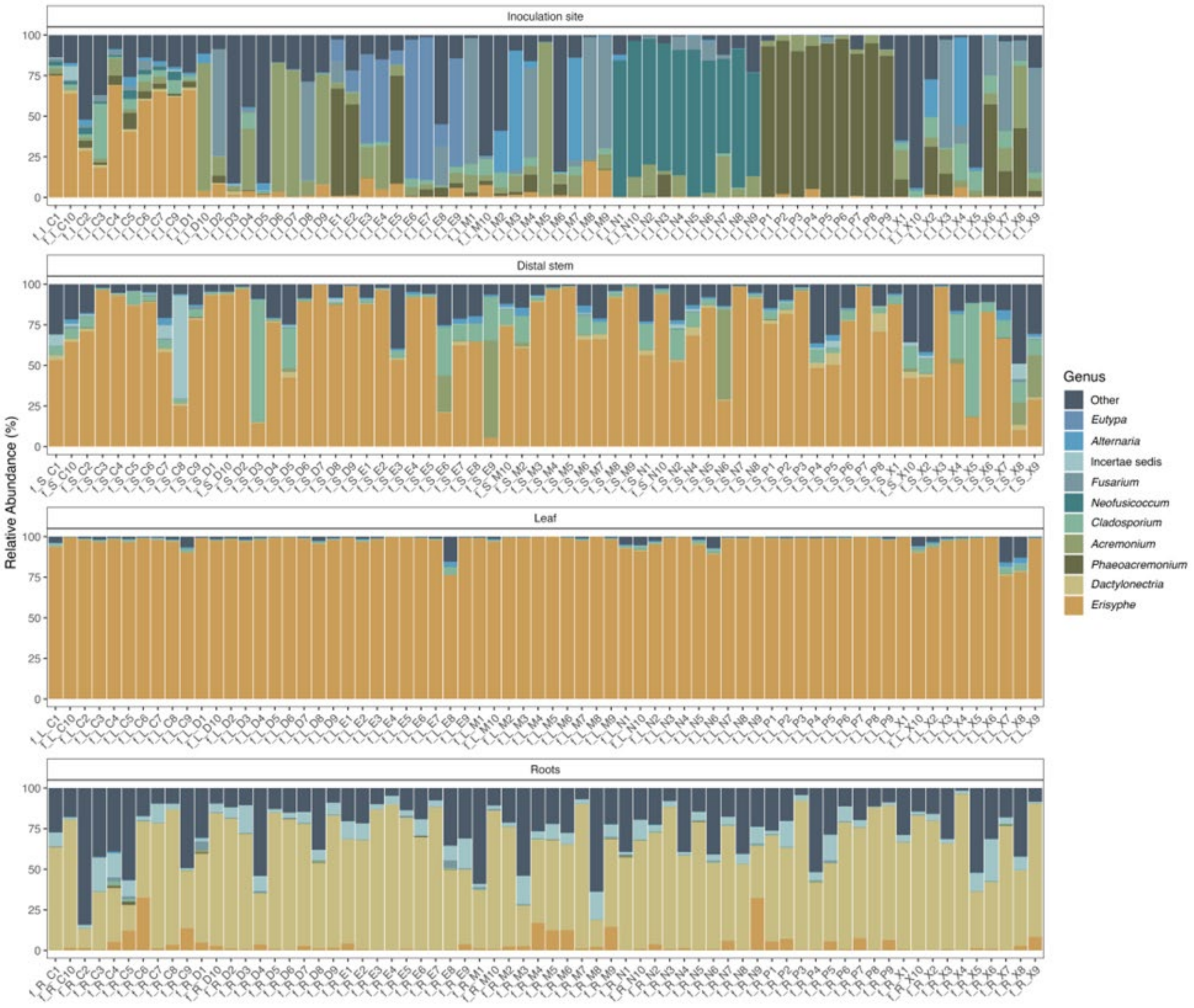

Supplementary Figure 5. Fungal relative abundance plots for all individual samples.

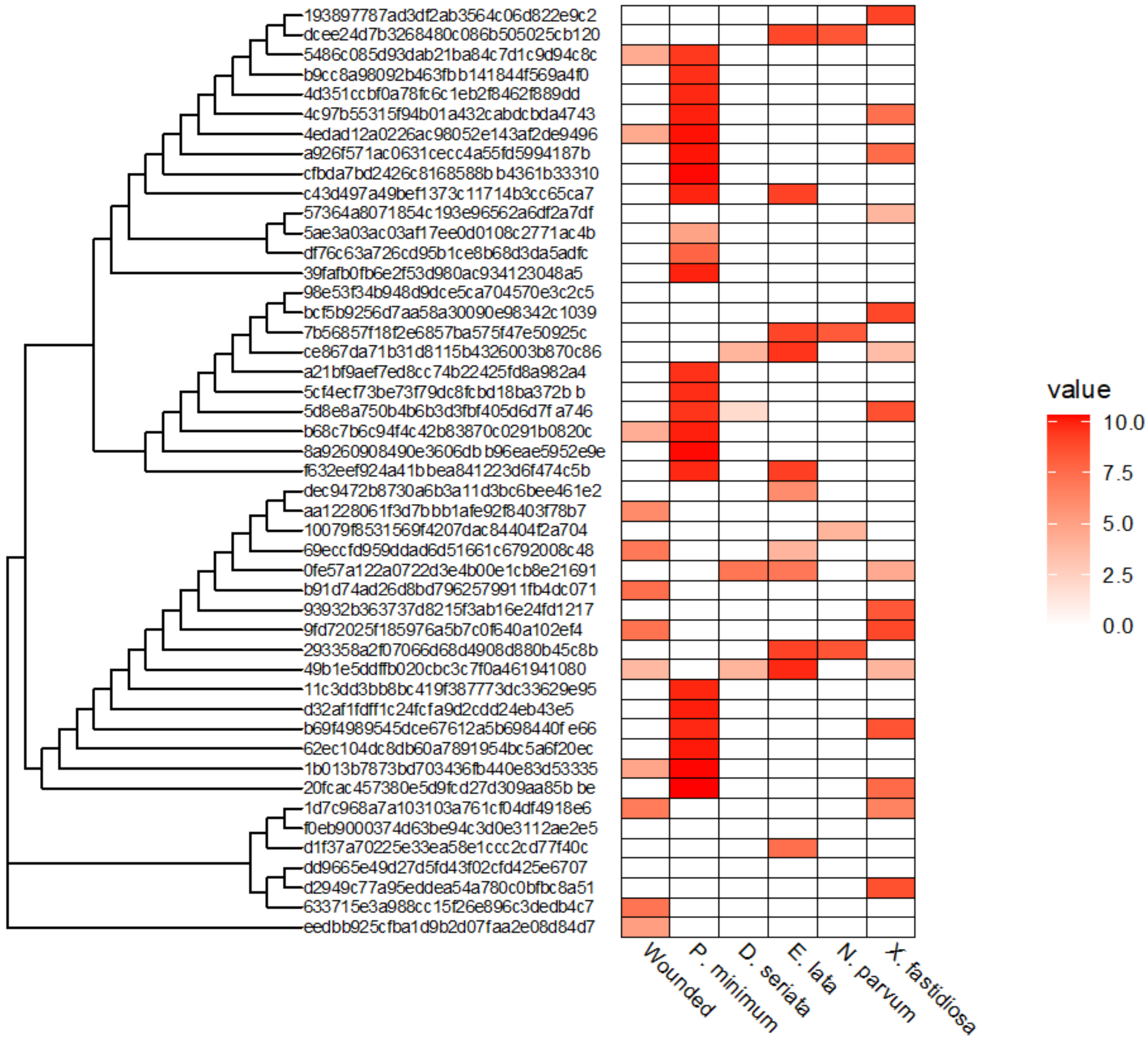

Supplementary Figure 6. Heatmap of normalized read counts for ASVs belonging to the *Phaeoacremonium* genus.

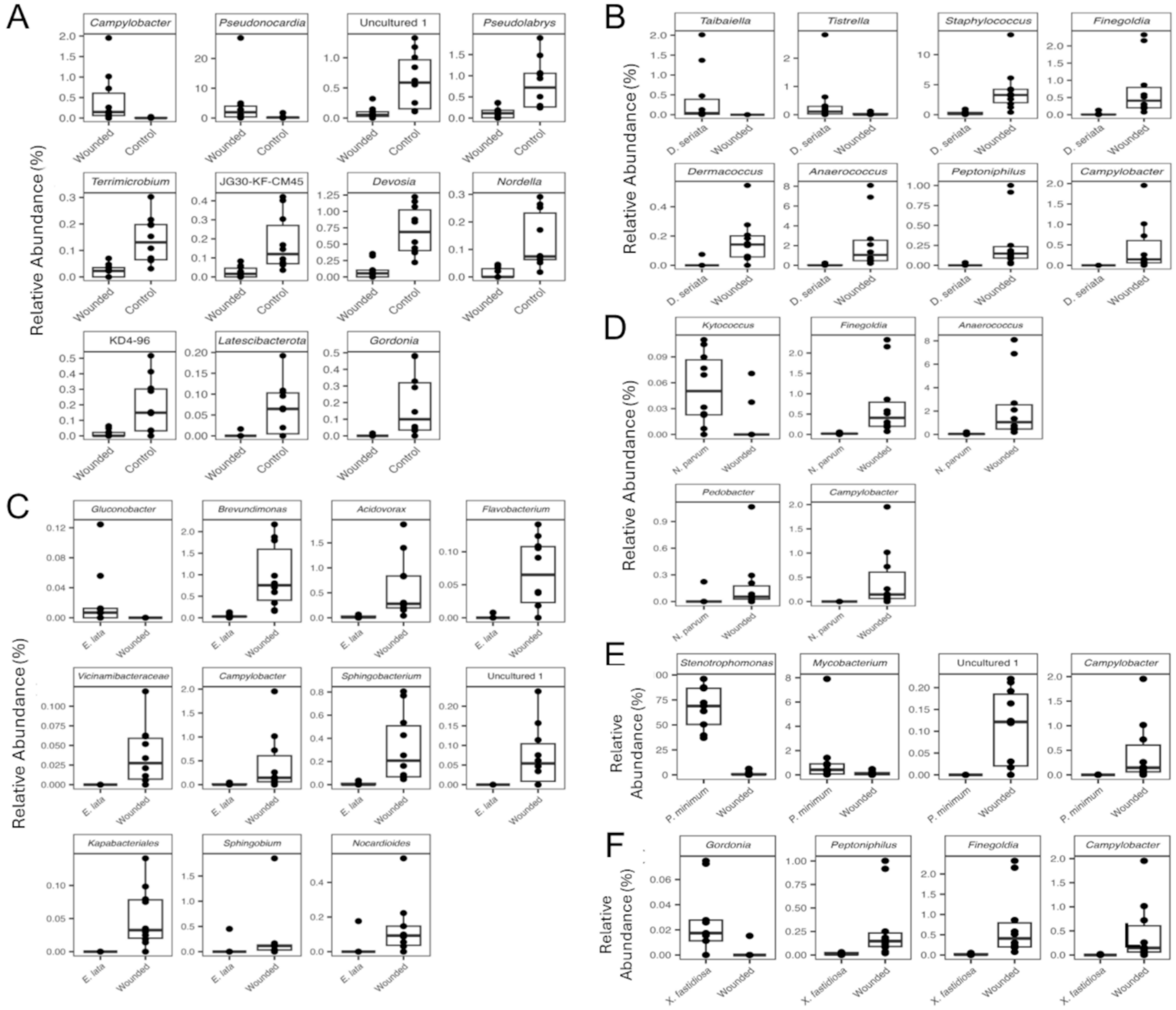

Supplemental Figure 7. Bacterial relative abundance for inoculation site samples for (A) wounded, (B) *D. seriata*, (C) *E. lata*, (D) *N. parvum*, (E) *P. minimum*, (F) *X. fastidiosa*.

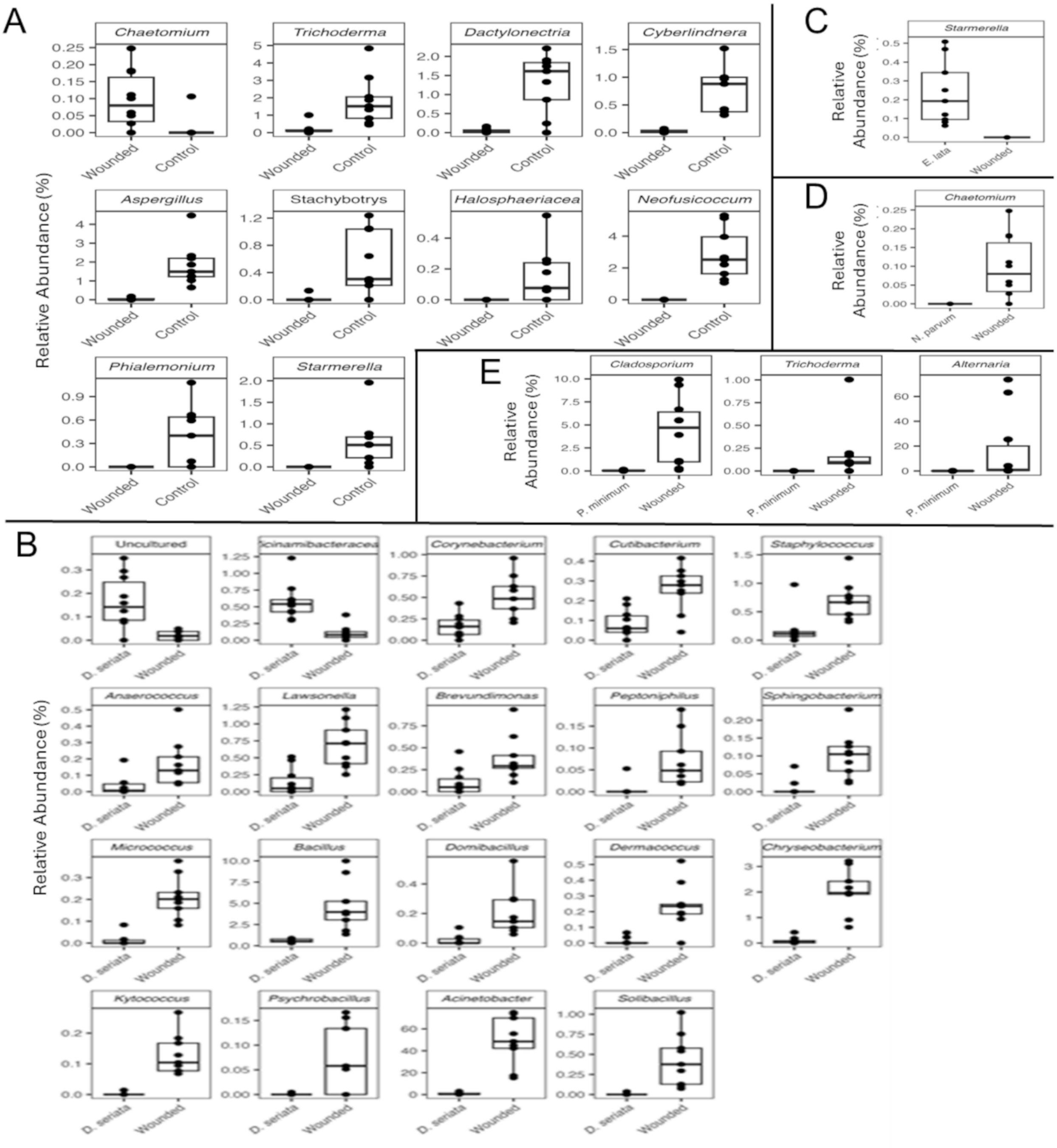

Supplemental Figure 8. Fungal relative abundance for inoculation site samples for (A) wounded, (B) *D. seriata*, (C) *E. lata*, (D) *N. parvum*, (E) *P. minimum*.

Supplementary Data 1. ANOVA tables showing significant differences in individual phenolic compound levels. For Tukey HSD separations among treatments: C = non-wounded control; D = *D. seriata*; E = *E. lata*; M = mock-inoculated; N = *N. parvum*; and X = *X. fastidiosa*. Note that no ANOVA statistics are given when a compound was unable to be quantified in a particular tissue, and only significant Tukey HSDs are provided.

	Inoculation Site			Distal Stem			Leaf			Root		
Compound	$F_{6, 57}$	p	Tukey HSD Seperations	$F_{6, 50}$	p	Tukey HSD Seperations	$F_{6, 59}$	p	Tukey HSD Seperations	$F_{6, 50}$	p	Tukey HSD Seperations
Hydroxycinnamatic acids												
caftaric acid	3.783	0.003	E>C,X	3.295	0.008	D>C,M,N,P						
caftaric acid derivative							3.119	0.010	D>M,N			
coutaric acid	4.096	0.002	E>C,M,D	1.944	0.092					1.558	0.183	
coutaric acid derivative	3.934	0.002	E>C; N>C,D,M,X; P>C,M	1.964	0.089		2.349	0.042	D>C,N	0.116	0.994	
feruloylquinic acid	6.026	0.000	E>C,D,M,X; N>C,M	0.745	0.616		1.330	0.258				
protocatechuic acid	2.099	0.067		3.368	0.007	D>M	1.805	0.114		1.364	0.251	
protocatechuic acid hexoside							5.154	0.000	C>M; D>M,N,P,X			
Flavan-3-ols and Procyanidins												
catechin	1.919	0.093		1.838	0.111		7.911	0.000	C>D,E,M,N,P,X	1.496	0.203	
gallocatechin										1.273	0.290	
epicatechin	2.006	0.080		1.939	0.093		1.379	0.238		0.692	0.657	
epicatechin gallate										1.312	0.273	
epigallocatechin										0.629	0.706	
epicatechin gallate	8.283	0.000	D>C; E>C; N>C; P>C; X>C	3.385	0.007	D>C,E,N,X	1.857	0.104		0.877	0.520	
procyanidin B1	3.862	0.003	E>C,P,X	1.915	0.097		1.419	0.223		0.919	0.491	

procyanidin B2	3.618	0.004	E>C; N>C	2.397	0.041	D>M	1.376	0.239		1.560	0.183
procyanidin B2 gallate	4.249	0.001	E>C; N>C	1.953	0.090		1.070	0.391		1.128	0.363
procyanidin B3	1.212	0.314		4.519	0.001	D>C,M,N,P,X					
procyanidin C1	1.684	0.142		2.219	0.056		0.682	0.665		0.792	0.581
procyanidin C2	2.124	0.064		0.084	0.543		1.579	0.169		1.888	0.105
unknown procyanidin dimer 1	2.938	0.014	none	1.742	0.131		2.635	0.025	none	1.704	0.144
unknown procyanidin dimer 2	4.032	0.002	E>C; N>C,D	1.866	0.105		2.092	0.068		1.531	0.192
unknown procyanidin dimer 3							1.812	0.112			
unknown procyanidin trimer gallate	3.700	0.004	E>C,D,X	1.837	0.111		0.609	0.722		1.360	0.253
Other Flavonoids											
afzelin	10.220	0.000	D>C; E>C,D,M,X; N>C; P>C; X>C	2.503	0.034	D>C,N				0.902	0.503
apigenin										1.545	0.188
astragalin	1.560	0.176		1.778	0.123		1.055	0.400		1.270	0.292
geinstein	5.747	0.000	D>C; E>C; N>C; P>C	8.328	0.000	D>C,E,N,P; X>C,E,N,P	1.924	0.092		1.165	0.343
kaempferol										0.526	0.786
kaempferol 3-O-glucoside	8.301	0.000	E>C; N>C,D,M,X; P>C	1.543	0.184		4.823	0.001	D>P,N,X	1.551	0.186
kaempferol 7-O-glucoside	1.757	0.125		1.618	0.162		7.604	0.000	N>C; X>C,D,E,M	2.021	0.084
kaempferol glucuronide	5.029	0.000	N>C,D,M,X	5.107	0.000	D>P,X; M>X	1.839	0.107		0.671	0.673

luteolin	10.472	0.000	E>C,D,M,X; N>C,D,M,X; P>C	4.236	0.002	D>C,E,N,P,X	1.804	0.114		0.630	0.705
naringenin	3.295	0.008	E>C; M>C; N>C; P>C	2.669	0.025	none	2.712	0.022	D>X	1.722	0.140
quercetin	8.042	0.000	E>C; N>C,D,M,X; P>C	4.093	0.002	D>C,E,N,P,X	5.759	0.000	D>C,E,M,N,P,X	0.737	0.623
quercetin glucoside	2.516	0.031	N>C	1.742	0.131		2.863	0.016	D>C	0.528	0.784
quercetin glucuronoide	13.852	0.000	D>C; E>C; N>C,M,X; P>C,D,M,X	1.723	0.135		1.353	0.249		2.134	0.069
quercitrin	2.605	0.027		1.045	0.408		1.291	0.276		0.774	0.595
rutin	2.960	0.014	C>N	2.684	0.025	P>C	2.105	0.066		0.412	0.867
unknown flavonoid										1.432	0.225
unknown flavonoid glycoside 1	8.899	0.000	D>C; E>C,M; N>C; P>C,M	2.103	0.069					1.761	0.131
unknown flavonoid glycoside 2	8.001	0.000	E>C; N>C,D,M,X; P>C	2.950	0.015	none				0.386	0.884
unknown flavonoid glycoside 3	6.839	0.000	D>C; E>C,M,X; N>C; P>C	3.971	0.003	D>C,E,N,P,X				1.390	0.241
Stilbenoids											
alpha-viniferin	11.045	0.000	D>C; E>C,M; M>C; N>C,M; P>C; X>C	5.415	0.000	D>C,E,M,N,P,X	4.827	0.001	X>C,E; N>C	1.507	0.200
delta-viniferin	7.807	0.000	E>C; N>C,D,M,X; P>C	3.287	0.008	D>N,P,X	2.911	0.015	D>X	1.684	0.149
epsilon-viniferin	12.492	0.000	D>C; E>C,M,X; M>C; N>C,M,X; P>C; X>C	3.696	0.004	D>C,E,N,P,X	4.787	0.001	D>C; M>C; N>C; X>C	1.042	0.412

miyabenol C	8.184	0.000	D>C; E>C; N>C; P>C,M,X	3.603	0.005	D>E,N,P,X				2.467	0.039	none
pallidol	8.053	0.000	D>C; E>C; N>C; P>C,M,X	3.578	0.005	D>C,E,N,P,X	1.079	0.385		1.798	0.123	
piceatannol	6.112	0.000	E>C,M; N>C,M; P>C	0.779	0.591					3.571	0.006	D>M,N,P,X
piceatannol derivative	1.293	0.275		3.198	0.010	D>E,N,P	1.893	0.097		2.329	0.050	
picied	14.292	0.000	N>C,M,X; P>C,D,E,M	3.433	0.007	P>C,X	0.740	0.620		1.504	0.200	
resveratrol										1.681	0.150	
stilbenoid trimer	4.248	0.001	N>C,M,X	2.788	0.020	C>N	2.111	0.065				
vitisin A	3.570	0.005	E>C; N>C	3.483	0.006	D>E,C,N,P,X				0.947	0.473	
vitisin B	4.657	0.001	D>N,X; P>C	0.964	0.459		6.038	0.000	M>C; N>C; P>C; X>C,E	1.049	0.408	
